# Supplementary material for: Genome-Wide Analysis of Aquaporins in Japanese Morning Glory (Ipomoea nil)
Source: Plants (Basel). 2023 Mar 30;12(7):1511. doi: 10.3390/plants12071511 (PMC10096635; doi:10.3390/plants12071511)
Supplement: Supplementary file 1 [file plants-12-01511-s001.zip › Figure S2.pdf]

```
INIL04g32909  CAATTTCCAATTTCAGATGAACATGGATCTTACGTTCGACCTACTAATTACGTGTTTTCC
INIL04g32910  ------------------------------------------------------------
XM_019307143  ------CCAATTTCAGATGAACATGGATCTTACGTTCGACCTACTAATTACGTGTTTTCC

INIL04g32909  ATCTACACGAAATTCCGAATTATTCATAAAAATTTAACCAGTTTCCTACTGCTCGCTGAC
INIL04g32910  ------------------------------------------------------------
XM_019307143  ATCTACACGAAATTCCGAATTATTCATAAAAATTTAACCAGTTTCCTACTGCTCGCTGAC

INIL04g32909  TGGGTGTCTTGGTAGGGAGAAGGAGAGTGATTGTTTAGGGTTTGGGGGAGAAGTAGGTAA
INIL04g32910  ------------------------------------------------------------
XM_019307143  TGGGTGTCTTGGTAGGGAGAAGGAGAGTGATTGTTTAGGGTTTGGGGGAGAAGTAGGTAA

INIL04g32909  TTCATATTCATTCAGCG-CCTCTGCCCAATACAAGCGTGGGTAGAGAGAGAGCTGTTCCA
INIL04g32910  ------------------------------------------------------------
XM_019307143  TTCATATTCATTCAGCG-CCTCTGCCCAATACAAGCGTGGGTAGAGAGAGAGCTGTTCCA

INIL04g32909  ACAATTCGAAGCTAAATTATTGATTCAGATCGGAGATTAGGAACAGATTGGAGTGAATAT
INIL04g32910  ------------------------------------------------------------
XM_019307143  ACAATTCGAAGCTAAATTATTGATTCAGATCGGAGATTAGGAACAGATTGGAGTGAATAT

INIL04g32909  GGGTGTGATTAAGGCAGCGATCGGCGATGCGGTGTTGACCTTCTTGTGGGTGTTCTCCGC
INIL04g32910  ------------------------------------------------------------
XM_019307143  GGGTGTGATTAAGGCAGCGATCGGCGATGCGGTGTTGACCTTCTTGTGGGTGTTCTCCGC

INIL04g32909  CTCCACGCTCGGCGTCAGCACTTCCGTTCTCGCCAAGCTGCTTGGAATTGCCCATCCAAT
INIL04g32910  ------------------------------------------------------------
XM_019307143  CTCCACGCTCGGCGTCAGCACTTCCGTTCTCGCCAAGCTGCTTGGAATTGCCCATCCAAT

INIL04g32909  GGCCGTTCTCTCCGTCACCACCGTCCTTTTTTTCATACTCCTGTTCGTTTTTGGTATCAT
INIL04g32910  ------------------------------------------------------------
XM_019307143  GGCCGTTCTCTCCGTCACCACCGTCCTTTTTTTCATACTCCTGTTCGTTTTTGGTATCAT

INIL04g32909  CAGTGATGCTCTCGGCGGCGCCAGCTTTAACCCTACCGGTATTGCTGCCTTTTATGCGGC
INIL04g32910  ------------------------------------------------------------
XM_019307143  CAGTGATGCTCTCGGCGGCGCCAGCTTTAACCCTACCGGTATTGCTGCCTTTTATGCGGC

INIL04g32909  TGGCCTCGGTGATGACTCTCTCATCTCGGCCGCCGTCCGTTTTCCAGCTCAGGTGGTTTA
INIL04g32910  ------------------------------------------------------------
XM_019307143  TGGCCTCGGTGATGACTCTCTCATCTCGGCCGCCGTCCGTTTTCCAGCTCAGGTGGTTTA

INIL04g32909  TTTGTTTGCGTTAAGGTTTTCTGTGAATTTTTCTGTTGGAAATGAGATTTATGCTGTTAA
INIL04g32910  ------------------------------------------------------------
XM_019307143  TTTGTTTGCGTTAAGGTTTTCTGTGAATTTTTCTGTTGGAAATGAGATTTATGCTGTTAA

INIL04g32909  ATATTGAAGGTTAGATGTTTAGGTGTTATCAGCATTGCCTTGGATCAGCCAATATACTAG
INIL04g32910  ------------------------------------------------------------
XM_019307143  ATATTGAAGGTTAGATGTTTAGGTGTTATCAGCATTGCCTTGGATCAGCCAATATACTAG

INIL04g32909  CAGTGCATCTTGTTCTAACTGGATTCAAAATAATTGGAAGACTGACAATTAGTGTTCATT
INIL04g32910  ------------------------------------------------------------
XM_019307143  CAGTGCATCTTGTTCTAACTGGATTCAAAATAATTGGAAGACTGACAATTAGTGTTCATT

INIL04g32909  GAGGGTGCAAAATGTTTTAGTTATGAATTGATACATAATAAACTAAATCATGATCA----
INIL04g32910  ------------------------------------------------------------
XM_019307143  GAGGGTGCAAAATGTTTTAGTTATGAATTGATACATAATAAACTAAATCATGATCATCCT

INIL04g32909  ------------------------------------------------------------
INIL04g32910  ------------------------------------------------------------
XM_019307143  TCTCAAATGACCTTGTGTCCTCCACATTACAAACATATGATTGCTGTGTGATCTTGACAG

INIL04g32909  ------------------------------------------------------------
INIL04g32910  -CTGCTGGTGCTGTTGGTGGTGCAATGGCGATTCTGGAAGTTATCCCTCCACATCACAAA
XM_019307143  GCTGCTGGTGCTGTTGGTGGTGCAATGGCGATTCTGGAAGTTATCCCTCCACATCACAAA
```

```
INIL04g32909  ------------------------------------------------------------
INIL04g32910  CACATGATTATTGGACCTTCTTTGAAGGTTGACCTTCACACCGGAGCCATTGCTGAGGGA
XM_019307143  CACATGATTATTGGACCTTCTTTGAAGGTTGACCTTCACACCGGAGCCATTGCTGAGGGA

INIL04g32909  ------------------------------------------------------------
INIL04g32910  GTCTTGACTTTCATAAGTTCCTTTATTGTTTTTCTTGTCATCCTGAAGGGTCCTAAAAAT
XM_019307143  GTCTTGACTTTCATAAGTTCCTTTATTGTTTTTCTTGTCATCCTGAAGGGTCCTAAAAAT

INIL04g32909  ------------------------------------------------------------
INIL04g32910  TCATTTGTCAAGAATTGGTTGCTTACCATACCAACAGTTGTCCTAGTAGTTGCTGGTTCT
XM_019307143  TCATTTGTCAAGAATTGGTTGCTTACCATACCAACAGTTGTCCTAGTAGTTGCTGGTTCT

INIL04g32909  ------------------------------------------------------------
INIL04g32910  AGCTATACAGGACCTTCCATGAATCCTGCTAATGTAAGTATAAATGATTTTTATTTCTTG
XM_019307143  AGCTATACAGGACCTTCCATGAATCCTGCTAATGTAAGTATAAATGATTTTTATTTCTTG

INIL04g32909  ------------------------------------------------------------
INIL04g32910  GTTTATATCTAAGGAGCATGGCATGATTAGTTTTTGTGTGCACACATTTTTTTTGATGTC
XM_019307143  GTTTATATCTAAGGAGCATGGCATGATTAGTTTTTGTGTGCACACATTTTTTTTGATGTC

INIL04g32909  ------------------------------------------------------------
INIL04g32910  AATTGATTGCTTGATTGTTCATCCTAAAAGAGAATTTGGTCTCTTATTAATTTTATTTTC
XM_019307143  AATTGATTGCTTGATTGTTCATCCTAAAAGAGAATTTGGTCTCTTATTAATTTTATTTTC

INIL04g32909  ------------------------------------------------------------
INIL04g32910  TGTGGTTCAGGTTTGATTAATGGCTTTACTACTGTTGCACTTGTATGCTCTCCATTCTCT
XM_019307143  TGTGGTTCAGGTTTGATTAATGGCTTTACTACTGTTGCACTTGTATGCTCTCCATTCTCT

INIL04g32909  ------------------------------------------------------------
INIL04g32910  GTTTTGTATCATTGTTCTCTTGGTTTGATTACTTTTTAATATGAGATCTGAACTTTGACT
XM_019307143  GTTTTGTATCATTGTTCTCTTGGTTTGATTACTTTTTAATATGAGATCTGAACTTTGACT

INIL04g32909  ------------------------------------------------------------
INIL04g32910  TTAAAGTGGATTTTGGTTGACATGTTACTGAAAGCCTGAAAATGTTGATGTGGAAAAGAA
XM_019307143  TTAAAGTGGATTTTGGTTGACATGTTACTGAAAGCCTGAAAATGTTGATGTGGAAAAGAA

INIL04g32909  ------------------------------------------------------------
INIL04g32910  TGTTGTTTAGTCTTTAAAATGCTCCTGCCAGGGTACTGAACAAATAATATAATATTGGTT
XM_019307143  TGTTGTTTAGTCTTTAAAATGCTCCTGCCAGGGTACTGAACAAATAATATAATATTGGTT

INIL04g32909  ------------------------------------------------------------
INIL04g32910  GAATTCAATTATTCAATTTTCAAAAGCCAGACTTGTATAAAAGGTAAATCCTAAAGTCAG
XM_019307143  GAATTCAATTATTCAATTTTCAAAAGCCAGACTTGTATAAAAGGTAAATCCTAAAGTCAG

INIL04g32909  ------------------------------------------------------------
INIL04g32910  GCATAATGGAGATGCCGTTATATCATGACTCCTCAAAGTGAAGATATTTATTGGCTGGTT
XM_019307143  GCATAATGGAGATGCCGTTATATCATGACTCCTCAAAGTGAAGATATTTATTGGCTGGTT

INIL04g32909  ------------------------------------------------------------
INIL04g32910  GTCCATACCTGATGGAAGCACTGCCACACATGTAGCGTTCTCTATCAGTAGTACAGTGGA
XM_019307143  GTCCATACCTGATGGAAGCACTGCCACACATGTAGCGTTCTCTATCAGTAGTACAGTGGA

INIL04g32909  ------------------------------------------------------------
INIL04g32910  AGATATTTAAAAGTTTTGATTGCCTACGGTCCAAACTCAAACCTTATCATCTGGGTGAAA
XM_019307143  AGATATTTAAAAGTTTTGATTGCCTACGGTCCAAACTCAAACCTTATCATCTGGGTGAAA

INIL04g32909  ------------------------------------------------------------
INIL04g32910  GTTAATTATATTTTTGAGCCTGTATTTCTCATGTTAGATTAGATGTTACCATAATCTAAG
XM_019307143  GTTAATTATATTTTTGAGCCTGTATTTCTCATGTTAGATTAGATGTTACCATAATCTAAG

INIL04g32909  ------------------------------------------------------------
INIL04g32910  AATGAAAGTGAAAGGTTTCCCTTTCTATCATTGTCTTTATACCCGTATTTGCTTGTTTAT
XM_019307143  AATGAAAGTGAAAGGTTTCCCTTTCTATCATTGTCTTTATACCCGTATTTGCTTGTTTAT
```

```
INIL04g32909  ------------------------------------------------------------
INIL04g32910  AATTATCATTTTCTAGGTTATTTTACTAAACTTGCCGCCTTCTTTTGTCATGCCCGGTAG
XM_019307143  AATTATCATTTTCTAGGTTATTTTACTAAACTTGCCGCCTTCTTTTGTCATGCCCGGTAG

INIL04g32909  ------------------------------------------------------------
INIL04g32910  GCTGGTAATCACATTACTTGAGCTACATTGACTGAAATCGTGGCATTATTATCATATTTG
XM_019307143  GCTGGTAATCACATTACTTGAGCTACATTGACTGAAATCGTGGCATTATTATCATATTTG

INIL04g32909  ------------------------------------------------------------
INIL04g32910  TTATAATAAATCACATTAATTATGTCCTATGCAATGCCTTTGATGATTCTTGATTGTTTC
XM_019307143  TTATAATAAATCACATTAATTATGTCCTATGCAATGCCTTTGATGATTCTTGATTGTTTC

INIL04g32909  ------------------------------------------------------------
INIL04g32910  GTGGCTGCGAAATTTCTTTACGTGATTGCCCTCTTTATCATCACCATAATTTACATTTTA
XM_019307143  GTGGCTGCGAAATTTCTTTACGTGATTGCCCTCTTTATCATCACCATAATTTACATTTTA

INIL04g32909  ------------------------------------------------------------
INIL04g32910  AAACCTGTTTTCCGATTTCCAATTTGGAGGAAACTGGATGTTGAAAGTGTGCTTTTTAAG
XM_019307143  AAACCTGTTTTCCGATTTCCAATTTGGAGGAAACTGGATGTTGAAAGTGTGCTTTTTAAG

INIL04g32909  ------------------------------------------------------------
INIL04g32910  TTTTTGGGTTAGACGTGTGTGCGCGCGGATATCCAGTTTTCCCTTTCATTTGTTTCTAGA
XM_019307143  TTTTTGGGTTAGACGTGTGTGCGCGCGGATATCCAGTTTTCCCTTTCATTTGTTTCTAGA

INIL04g32909  ------------------------------------------------------------
INIL04g32910  TGGCTGGAAATTTGTATTCTTTCTATCCTTCTTTCTCTTCTAGTAATATGATGGTATTTT
XM_019307143  TGGCTGGAAATTTGTATTCTTTCTATCCTTCTTTCTCTTCTAGTAATATGATGGTATTTT

INIL04g32909  ------------------------------------------------------------
INIL04g32910  GTATATGATTAAATACATTTCACACAGACAATTATATTCCTCTGTTTGTCATTTAATTTC
XM_019307143  GTATATGATTAAATACATTTCACACAGACAATTATATTCCTCTGTTTGTCATTTAATTTC

INIL04g32909  ------------------------------------------------------------
INIL04g32910  CTGATTGAGTATCAAACTTCGAGGCTGTGATGTAAAGCTGGTTCCTGAATTTACGTTTGC
XM_019307143  CTGATTGAGTATCAAACTTCGAGGCTGTGATGTAAAGCTGGTTCCTGAATTTACGTTTGC

INIL04g32909  ------------------------------------------------------------
INIL04g32910  AGGCATTCGGCTGGGCTTACATAAATAACTGGCACAATACGCGGGAGCAGTTTTATGTTT
XM_019307143  AGGCATTCGGCTGGGCTTACATAAATAACTGGCACAATACGCGGGAGCAGTTTTATGTTT

INIL04g32909  ------------------------------------------------------------
INIL04g32910  ACTGGATTTGCCCCTTCATCGGAGCAATATTGGCGGCGTGGATGTTCCGAGCTATATTTC
XM_019307143  ACTGGATTTGCCCCTTCATCGGAGCAATATTGGCGGCGTGGATGTTCCGAGCTATATTTC

INIL04g32909  ------------------------------------------------------------
INIL04g32910  CACCGCCAGTGAAGCCAAAGGCGAAGAAGAACTAAAAAGACAGTTCAATAGATTGATTGT
XM_019307143  CACCGCCAGTGAAGCCAAAGGCGAAGAAGAACTAAAAAGACAGTTCAATAGATTGATTGT

INIL04g32909  ------------------------------------------------------------
INIL04g32910  CCTGGTTTTAGATCGTTGCTATATAGTTATGATAGCTAAGTGGACGTCTAGTAAATGGAC
XM_019307143  CCTGGTTTTAGATCGTTGCTATATAGTTATGATAGCTAAGTGGACGTCTAGTAAATGGAC

INIL04g32909  ------------------------------------------------------------
INIL04g32910  TCCGTGTTTTAGATTGATTCATGTAAGTTGATTGAACTGGAATTTTTATATCCAATAAAT
XM_019307143  TCCGTGTTTTAGATTGATTCATGTAAGTTGATTGAACTGGAATTTTTATATCCAATAAAT

INIL04g32909  ------------------------------------------------------------
INIL04g32910  ATCCTTCCCACAT-----------------------------------------------
XM_019307143  ATCCTTCCCACATTTATAATGCTTTGATAGTCATTGATATGGAGTAATGGTATTAGCCTT

INIL04g32909  --------
INIL04g32910  --------
XM_019307143  GTTTTGCA
```

Figure S2 Alignment of genome sequences of INIL04g32909, INIL04g32910 and XM_019307143.

Shown is a genome sequence alignment of INIL04g32909, INIL04g32910 and XM_019307143. Exons of INIL04g32909 are shown in yellow, exons of INIL04g32910 in gray, and exons of XM_019307143 in green. Bold letters indicate start and end codons.
