# Supplementary material for: Genome-Wide Analysis of Aquaporins in Japanese Morning Glory (Ipomoea nil)
Source: Plants (Basel). 2023 Mar 30;12(7):1511. doi: 10.3390/plants12071511 (PMC10096635; doi:10.3390/plants12071511)
Supplement: Supplementary file 1 [file plants-12-01511-s001.zip › Figure S3.pdf]

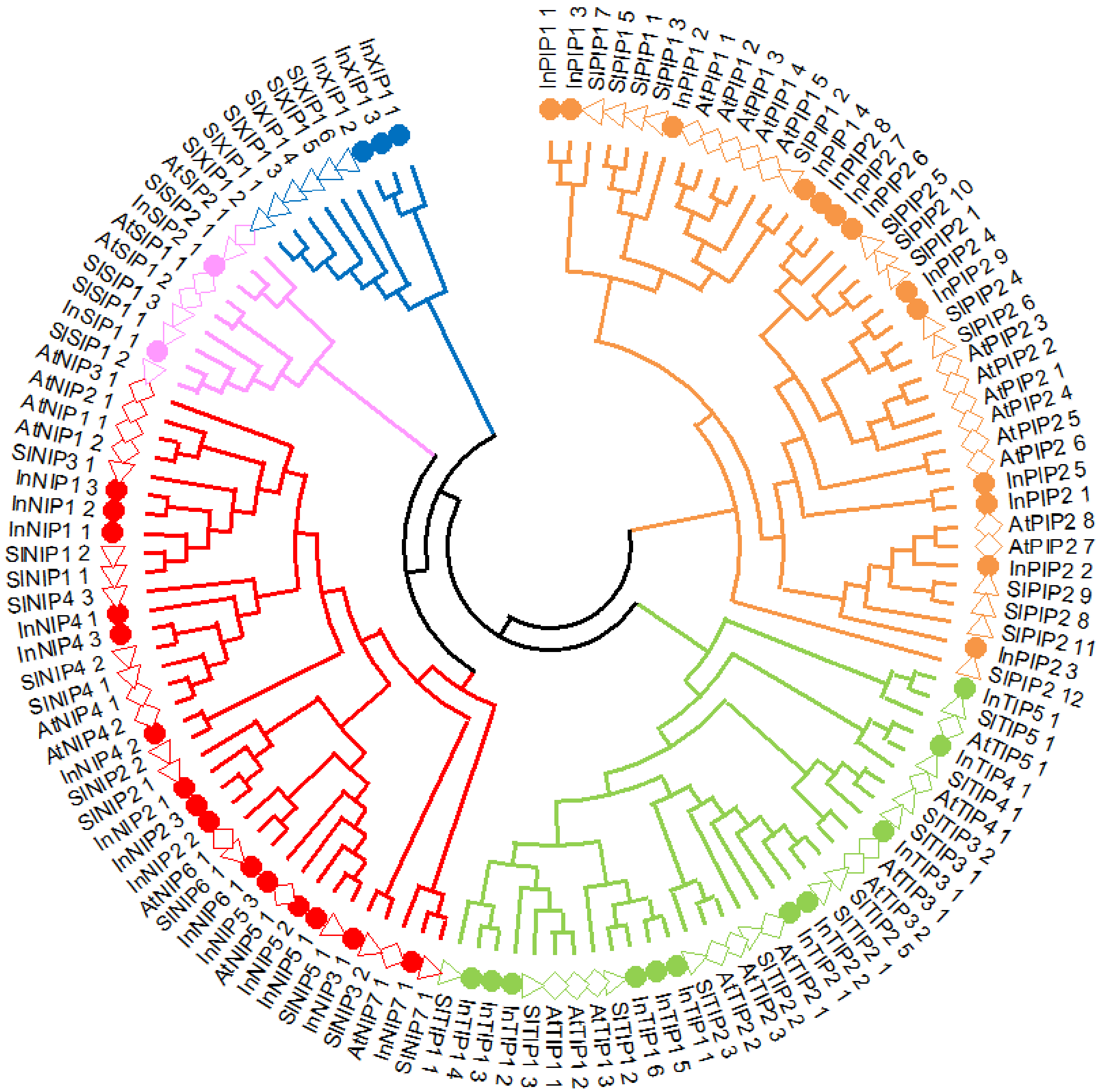

Figure S3. Phylogenetic trees of aquaporins of Arabidopsis, tomato and Japanese morning glory. Phylogenetic tree is generated by the neighbor-joining method derived from a CLUSTAL alignment of the aquaporins amino acid sequences of *Arabidopsis* (◇, [11]]), tomato (△, [13]) and Japanese morning glory (●, this study).
