# Supplementary material for: Genome-Wide Analysis of Aquaporins in Japanese Morning Glory (Ipomoea nil)
Source: Plants (Basel). 2023 Mar 30;12(7):1511. doi: 10.3390/plants12071511 (PMC10096635; doi:10.3390/plants12071511)
Supplement: Supplementary file 1 [file plants-12-01511-s001.zip › Figure S4.pdf]

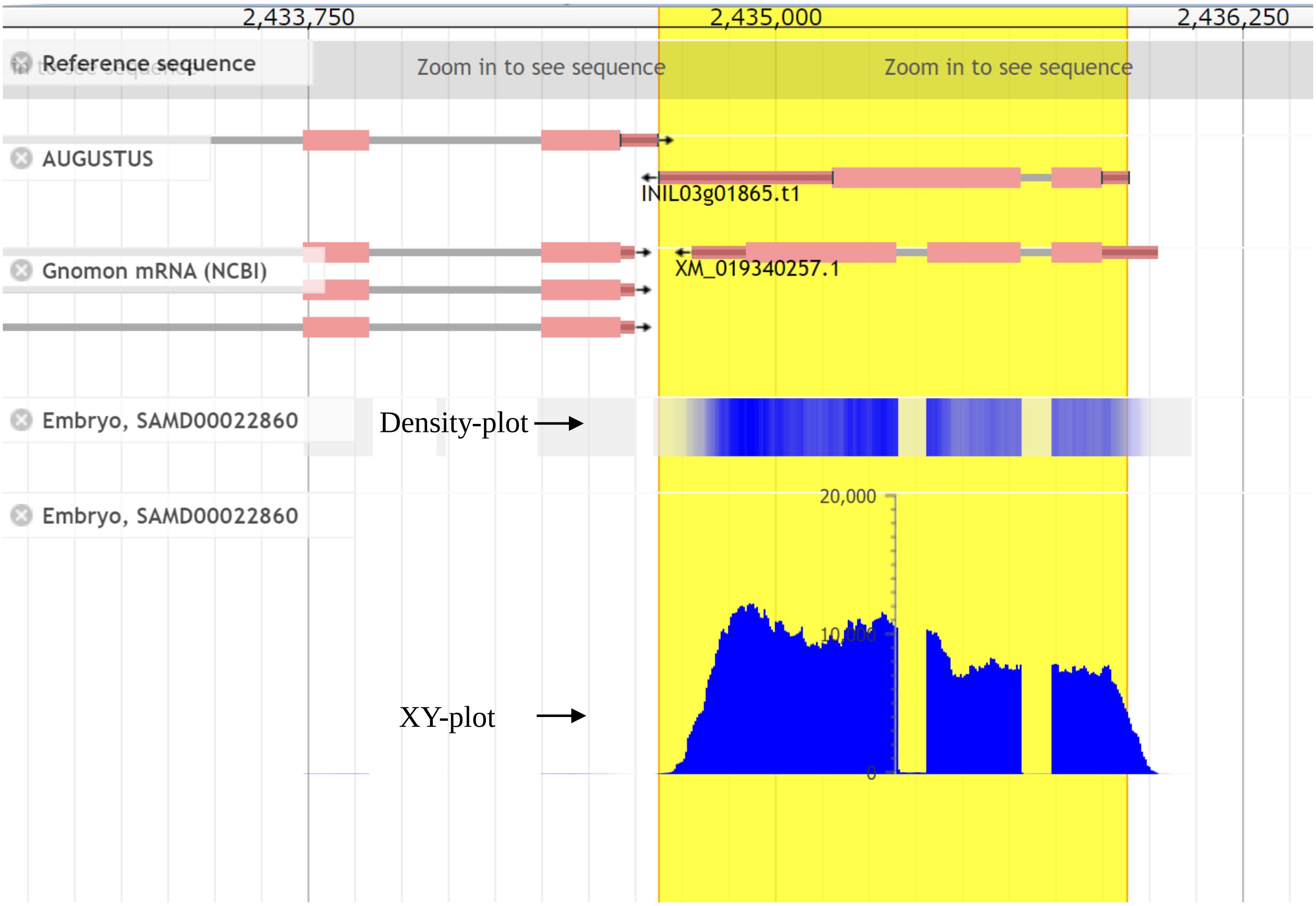

FigS4. Schematic diagram of the gene structure and gene expression of InTIP3;1 on JBrowse. The gene structures are information from AUGUSTUS annotation and Gnomon annotation, respectively. The graphs show the density-plot and XY-plot in the embryo, respectively; the density-plot shows that the darker the blue color, the higher the expression, and the XY-plot shows that the higher the expression, the more mountainous the plot.
