# Supplementary material for: Genome-Wide Analysis of Aquaporins in Japanese Morning Glory (Ipomoea nil)
Source: Plants (Basel). 2023 Mar 30;12(7):1511. doi: 10.3390/plants12071511 (PMC10096635; doi:10.3390/plants12071511)
Supplement: Supplementary file 1 [file plants-12-01511-s001.zip › Figure S5.pdf]

```
InNIP1;1  -----------------------------MAESNGNHVVALDLKDE---RSNLDDSSSPG
InNIP1;2  -----------------------------MAGNHGEHSVALDISDDHHHHHTADDASETT
InNIP1;3  -----------------------------MARNHGDHSVALDISDD--HHHTVDDASETT
InNIP2;1  --------------------------------MENEDGKGNNVNGLVSVENPKSQSRLGR
InNIP2;2  --------------------------------MENEDGKGNNVNGLVSVENPKSQSRLGR
InNIP2;3  --------------------------------MENEDGKGNNVNGLVSVENPKSQSRLGR
InNIP3;1  ---------------------------------------------MEEETKHKHVFHSRE
InNIP4;1  --------------------------------------------MTKCDDRVEEEAISNM
InNIP4;2  -------MAAEISSVANGNRNATTLDIRDDDVLCRPRNFLPCTCSDMAFKKYAEAAAVSS
InNIP4;3  -----------------------------MAAKESIVTIEEGRTQSGNQERDEHSVEGRK
InNIP5;1  -MPEPETETPTASAPATPGTPAPLFTSLHVDSLSYDRKSMPRC-KCFPLDAPTWGAPHTC
InNIP5;2  MPDCEDGTLTAASAPATPGTPTPLFTSFRVDSLSYDRKSMPRCNKCLPLDVPTLGTPHTC
InNIP5;3  ---MPEYEAARAPPATSGGTPTPFLTSVRIDSPPCDRN----------------------
InNIP6;1  ------------------------------------------------------------
InNIP7;1  ------------------------------------------------------------

                                                                H2
InNIP1;1  CD--FIFTLPFIQKLIAEMFGTYFLIFAGCGAVVVNAEKG-SITFPGISIVWGLVVMVMV
InNIP1;2  CNNNFIFTLPFIQKVIAEIFGTYFLIFAGCGAAAVNADKG-TVTFPGVAIVWGLAAVAMI
InNIP1;3  CNNNFIFTLPFIQKVIAEIFGTYFLIFAGCGAAAVNADKG-TVTFPGVAIVWGLAAVAMI
InNIP2;1  LFQRQCYPPHFLNKVVAEVIATYLLVFVTAGSAAISASDAHRVSQLGASVAGGLIVTVMI
InNIP2;2  LFQRQCYPPHFLNKVVAEVIATYLLVFVTAGSAAISASDAHRVSQLGASVAGGLIVTVMI
InNIP2;3  LFQRQCYPPHFLNKVVAEVIATYLLVFVTAGSAAISASDAHRVSQLGASVAGGLIVTVMI
InNIP3;1  FPREASAFISAFQKIVAELVGTYIFIFVGCGSALVDREKE--LTMVGIALAWGLPLMALI
InNIP4;1  EQGTATGTGTSTFRNAAEVVGVYFIIFAGCGSVAVNKLYGGTVTFPGICVTWGLIVMVMV
InNIP4;2  SAAGYSDFMRFMQKLIAEFMGTYLLLFAGFAALLTNKDLS----LPVTAMLWGMDVMIML
InNIP4;3  TFCTSSAVVVIIQKMIAETIGTYFLIFIGCGSVAVNKIYG-SVTFPGVSVAWGLVIMVMV
InNIP5;1  LTDFPAPDISLTRKVGAEFVGTFILIFAATAGPIVNQKYNGAESLIGNAACAGLAVMIII
InNIP5;2  LTDWPAPDVSLTRKLGAEFVGTFILIFAATAGPIVNQKYNGVETLIGNAACAGLAVMIVI
InNIP5;3  -------------PVGAEFVGTFILIFTSTAAPIVNQKYNGAETLIGNAACAGLAVMSII
InNIP6;1  ----------------------MILIFAGTATAIVNQKTQGAETLLGLAGSTGLAVMIVI
InNIP7;1  --------------MMAEALGTYILVFCICGIISNMQLMGVQVGLLEYAATASLTVIVVV

InNIP1;1  YSVGHISGAHFNPAVTIAFASCKRFPFKQVPLYLCAQILGATLASGTLRLLFHGTHDHFA
InNIP1;2  YSVGHISGAHFNPAVTIAFATCRRFPCAQVPAYILAQMIGATAASGTLRLIFNGEHDHFV
InNIP1;3  YSVGHISGAHFNPAVTIAFATCKRFPCAQVPAYILAQMIGATAASGTLRLIFNGEHDHFV
InNIP2;1  YAVGHISGAHMNPAVTFAFAAFRHFPWRQVPFYAAAQVTGATSAAFTLRVILNPIRRIGT
InNIP2;2  YAVGHISGAHMNPAVTFAFAAFRHFPWRQVPFYAAAQVTGATSAAFTLRVILNPIRRIGT
InNIP2;3  YAVGHISGAHMNPAVTFAFAAFRHFPWRQVPFYAAAQVTGATSAAFTLRVILNPIRRIGT
InNIP3;1  YTLGHVSGAHFNPAVTIAFGTSGRLPLLQVPMYVVCQLLGSTLACLTLKALFNRQNDIKP
InNIP4;1  YTLGHVSDGHFNPAVTLTNAIFARFSWKLVPVYIVAQLIGSILASSTLAMIFDITPEAFF
InNIP4;2  YTVGHLSGAHFNPAVTLAFASCKRFPWRHVPAYIIAQVLAATLATGTVRLMFSAEEDHFL
InNIP4;3  YSVGHISGAHFNPAVTVTFALFRKFPWRQVPLYIFAQLTGSIVASGTLYFLLEVNPKAFF
InNIP5;1  LSTGHISGAHLNPSLTIAFAALRHFPWAQVPAYIAAQVSASICASFALKGVFHPFLSGGV
InNIP5;2  LSTGHISGAHLNPSLTIAFAAFRHFPWAQVPAYILAQVSASICASFCLKAVFHPIMSGGV
InNIP5;3  LSTGHISGAHLNPSLTIAFAAFRHFPWSQVPAYIVAQVSASISASLAIKAIFHPFMSGGV
InNIP6;1  LSTGHISGAHLNPAVTIAFAALKHFPWKHVPVYVGTQVMASLGAAFILKAVFHPIMGGGV
InNIP7;1  FSIGPISGAHINPSVTLAFASLGPFPWPKVPFYIMAQVGGSVFATVSSRLIYGVEYEHMM
```

```
                                                                    H5
InNIP1;1  GTLPQGS---DSQSLVIEFIITFYLMFVISGVATDNRAIG-ELAGLAVGATVLLNVMFAG
InNIP1;2  GTAPAGS---NLQSLIIEFIITFYLVFVISSVATDTRAVG-ELAGVVIGATILLNVMFTG
InNIP1;3  GTAPAGS---DLQSLVIEFIITFYLMFVISSVVTDTRAVG-ELAGLVIGATILLNVMFTG
InNIP2;1  TTPSGNNY---VQALITEIVVTFSMMFVTSAVATDTKAIGELAGIAVGSAVCITSIFAGP
InNIP2;2  TTPSGNNY---VQALITEIVVTFSMMFVTSAVATDTKAIGSWLALQWVRPCALLQSLLGN
InNIP2;3  TTPSGNNY---VQALITEIVVTFSMMFVTSAVATDTKAIGSWLALQWVRPCALLQSLLLY
InNIP3;1  TLTQYNTPTTDLEAITWEFLMTFILMFVICGAASDDRANK-EVAGVAIGVTLLFEVLVAG
InNIP4;1  GTLPAGS---NLQSFVVEIIISFLLMFVISSISTDDRATVGDLSGVVIGMTILLNVLVAG
InNIP4;2  GTVPAGS---DMQSLILEFLITFYLMFAVSGVATDKRGVS-EFTGLSVGAVIAINSIIAG
InNIP4;3  GTAPVGS---DIQSFIIEFIMSFLLMFVISGVATDNRAIG-ELAGIAIGMTILICVLVAG
InNIP5;1  TVPSVSTG----QAFALEFLITFNLLFVVTAVATDTRAVG-ELAGIAVGATVMLNILVAG
InNIP5;2  TVPSVNLG----QAFGLEFLITFNLLFVVTAVATDTRAVG-ELAGIAVGATVMLNILVAG
InNIP5;3  TVPSVAAG----QAFALEFLITFNLLFVVTAVATDSRAVG-ELAGVAVGATVMLNILIAG
InNIP6;1  TVPSGNYI----QAFALEFIISFILMFVITAVSTDTRAVG-ELAGIAVGATVMVNILIAG
InNIP7;1  TRPLHGCN----AAFWVEFLATFIILFLASSLSSDPQFTG-KLAGFVVGVCIGLGVLISG

                                      LE1   LE2
InNIP1;1  PISG----------------------ASMNPARSLGPAIVSKKYTG-IWIYLLGPTAGAIA
InNIP1;2  PITG----------------------ASMNPARSLGPAIVSNRYRG-IWIYMLGPTAGAIA
InNIP1;3  PITG----------------------ASMNPARSLGPAIVSNRYRG-IWIYMLGPTAGAIA
InNIP2;1  ISGG-----------------------SMNPARTIGPAIASNDYKG-IWIYIVGPVCGTLL
InNIP2;2  YRTRNPVNLTRLVLSETRYEQADIGGSMNPARTIGPAIASNDYKG-IWIYIVGPVCGTLL
InNIP2;3  PPHR---------------------------------------------------------
InNIP3;1  PITG----------------------ASMNPARSIGPAVVSGVYKN-QWVFVVAPIVGAMA
InNIP4;1  PISG----------------------ASMNPARSIGPAIVKHTYRG-LWVYILGPIIGMVA
InNIP4;2  PISG----------------------ASMNPARTLGPAIISNRYKS-IWVYMLGPTAGATA
InNIP4;3  PVSG----------------------ASMNPARTLGPAIVMHVYKS-IWVYMVGPMMGTIS
InNIP5;1  PSSG----------------------GSMNPVRTLGPAVAAGNYKS-IWIYLVAPTLGAIA
InNIP5;2  PSSG----------------------GSMNPVRTLGPAVAAGNYDS-IWIYLVAPTLGALA
InNIP5;3  PSSG----------------------GSMNPVRSIGPAVAAGNYKS-IWIYLIAPTLGALA
InNIP6;1  QTTG----------------------ASMNPVRTLGPAIAAGNYRA-IWIYLTAPILGALA
InNIP7;1  PVSG----------------------GSMNPARSLGPAIASWSFDSDLWIYFVAPTTGAVA

InNIP1;1  GAWVYNTIRFTDKPLREITKSGSFLRSKGSSSRNGSK-------------
InNIP1;2  EPNVA---------------------------------------------
InNIP1;3  GAWVYNILRLKNKP------------------------------------
InNIP2;1  GAWCYSFIRLTDKPTHAVQPQHLPSFQLRRMKNDNVEHTAADVRDPLNAL
InNIP2;2  GAWCYSFIRLTDKPTHAVQPQHLPSFQLRRMKNDNVEHTAADVRDPLNAL
InNIP2;3  ------------QATHAVQPQHLPSFQLRRMKNDNVEHTAADVRDPLNAL
InNIP3;1  ATLVYSLLRPTKNAVHSEETTKSVVYNDLYSHSQVV--------------
InNIP4;1  GAFVYNVLKLTHKPASDSSRTRSFLKQLRS--------------------
InNIP4;2  GVWFYNAVRLQDKPSDEEIIKSQIFPQDCPTN------------------
InNIP4;3  GGFTYNLIRFTEKPLSELTRSGPCSL ------------------------
InNIP5;1  GAAIYTVVKLREEVEPTSRPRSFRR-------------------------
InNIP5;2  GAAAYTVVKLQEGEVDEPLRQHRSFRR
InNIP5;3  GASIYTLLKSKGSRT-----------------------------------
InNIP6;1  GAGVYSAVKLPDEDGENQQKPSVEHSFRR---------------------
InNIP7;1  GVFLYRLLRLQGWTCKPADASPNTPLHNNYNNPSN---------------
```

Figure S5: Alignment of AA sequences of InNIP subfamily members.

Shown is an AA sequence alignment of all InNIP. Black lines above the alignment indicate predicted transmembrane domains. The two conserved NPA motifs are shown in bold letters and marked in yellow. Residues comprising the ar/R filter are marked in blue and labelled H2, H5, LE1 and LE2. Residues occupying conserved positions one to five (from N- to C-terminus P1 to P5) are marked in green.
