# Supplementary material for: Genome-Wide Analysis of Aquaporins in Japanese Morning Glory (Ipomoea nil)
Source: Plants (Basel). 2023 Mar 30;12(7):1511. doi: 10.3390/plants12071511 (PMC10096635; doi:10.3390/plants12071511)
Supplement: Supplementary file 1 [file plants-12-01511-s001.zip › Figure S6.pdf]

```
InPIP1;1  -MEHREEDVRLGANKFPEKQAIGTAAQ-DKDYTEAPPTALFEPSELSSWSFYRAGIAEFI
InPIP1;2  MAESKEEDVKLGANKFRETQPLGTAAQTDKDYKEPPPAPLFEPGELTSWSFYRAGIAEFV
InPIP1;3  -MEHREEDVNLGANKFSERQAIGTAAQQDKDYSEPPAAPLFEEGELMSWSFYRAGIAEFM
InPIP1;4  -MEGKEEDVMVGANKFSERMPLGTSAQ-GKDYKEAPPAPLFEAGELHSWSFWRAGIAEFI
InPIP2;1  ----------------MTKDMAEPGSFSTKDYHDPPPAPLIDAEELTKWSFYRAVIAEFI
InPIP2;2  ---------MGRKDVTRVSEEGQAPHHVGKDYVDPPPAPLLDFSELKLWSFYRAVIAEFI
InPIP2;3  --------------------------MSTKDYVEPPPAALLDMDELKKWSFYRAVIAEFV
InPIP2;4  ----------------MAKDIEGTAEHSAKDYHDPPPAPLIDFDELTKWSFYRAAIAEFI
InPIP2;5  --------------MAKGTAAEPAGSFTAKDYQDPPPAPLIDPEELTQWSFYRALIAEFI
InPIP2;6  ---------------MGKDVEVGNEYS-LKDYQDPPPAPFIDPEELGQWSFYRAIIAEFV
InPIP2;7  ---------------MAKDVEVGTEYAAPKDYQDPPPAPLIDPEELGKWSFYRAIIAEFI
InPIP2;8  ---------------MAKDVEGATEYAAPKDYQDPPPAPLIDPEELGKWSFYRAIIAEFI
InPIP2;9  ------------MTKDVEAVPEQPAEFSAKDYHDPPPSPLIDFDELTKWSFYRAVIAEFI

                                                       H2
InPIP1;1  ATFLFLYITILTVMGVSKSDS-------KCKTVGIQGIAWAFGGMIFALVYCTAGISGGH
InPIP1;2  ATFLFLYITILTVMGVKRSDG-------ICKSVGIQGIAWAFGGMIFALVYCTAGISGGH
InPIP1;3  ATFLFLYITVLTVMGVVKSES-------KCSTVGIQGIAWAFGGMIFALVYCTAGISGGH
InPIP1;4  ATFLFLYITVLTVMGYSRSKS-------KCNTVGVQGIAWAFGGMIFALVYCTAGISGGH
InPIP2;1  ATLLFLYITVLTVIGYKSETA--DPKADPCGGVGILGIAWAFGGMIFILVYCTAGISGGH
InPIP2;2  ATLLFLYVTVATVIGHKEQQ----TAAGPCDGVGLLGIAWAFGGMIFVLVYCTAGISGGH
InPIP2;3  ATLLFLYVSVATVIGTK-------ATPDPCKGAGLLGISWVFGGMIFVLVYCTAGISGGH
InPIP2;4  ATLLFLYVTVLTVIGYSHQTDLKAG-GQDCGGVGILGIAWAFGGMIFILVYCTAGISGGH
InPIP2;5  ATLLFLYVTVLTVIGHS-------KEADACNGVGVLGIAWAFGGMIFILVYCTAGISGGH
InPIP2;6  ATLLFLYVTVLTVIGYKSQTDPHVTGTDACGGVGILGIAWAFGGMIFILVYCTAGISGGH
InPIP2;7  ATLLFLYVTVLTVIGYKSQTDPNVKGTDECGGVGILGIAWAFGGMIFILVYCTAGISGGH
InPIP2;8  ATLLFLYVTVLTVIGYKSQTDPNVKGTDECGGVGILGIAWAFGGMIFILVYCTAGISGGH
InPIP2;9  ATLLFLYVTVLTVIGYKHQTDPKAG-GQDCGGVGILGIAWAFGGMIFILVYCTAGISGGH

InPIP1;1  INPAVTFGLFLARKVSLTRLVYYIVMQCLGAICGAGVVKGFGKTLYNSKGGGANVVNPGY
InPIP1;2  INPAVTFGLFLARKLSLTRALFYMVMQCLGAICGAGVVKGFQKTEYMFYKGGTNSVAHGY
InPIP1;3  INPAVTFGLFLARKLSLTRAVFYMVMQCLGAICGAGVVKGFGKTVYNGKGGGANVVAPGY
InPIP1;4  INPAVTFGLFLARKLSLTRAIFYIVMQCLGAICGAGVVKGFQPSLYVSNGGGANVVAHGY
InPIP2;1  INPAVTFGLFLARKVSLIRAVMYMVAQCLGAICGVGLVKAFQKAYYTRYGGGANQLAHGY
InPIP2;2  INPAVTFGLFLARKVSLVRAVLYMVAQCLGGICGVGLVKALMKDYYNRQGGGANSVGHGY
InPIP2;3  INPAVTFGLFLARKVSLIRAVAYMVAQCLGAVVGVGLVKALTKDFFDTVGGGANTVQPGF
InPIP2;4  INPAVTFGLFVGRKVSLVRAVVYMIAQCLGAICGVGLVKAFQKAYFNRYGGGANMLQPGY
InPIP2;5  INPAVTFGLFLARKVSLVRAVMYMAAQCLGAICGVGLVKAFQSSYYTRYGGGANKMAEGY
InPIP2;6  INPAVTFGLFLARKVSLVRAIMYMVAQCLGAICGCGLVKAFQKAYYVRYGGGANELADGY
InPIP2;7  INPAVTFGLFLARKVSLVRAVMYMVAQCLGAICGCGLVKAFQKAYYVRYGGGANGLQDGY
InPIP2;8  INPAVTFGLFLARKVSLVRAVMYMVAQCLGAICGCGLVKAFQKAYYVRYGGGANGLQDGY
InPIP2;9  INPAVTFGLFLARKVTLPRAVLYMIAQCLGAICGVGLVKAFQKSFYNRYGGGANEMAAGY

                                                         H5     LE1
InPIP1;1  TKGDGLGAEIVGTFVLVYTVFSATDAKRNARDSHVPVLAPLPIGFAVFLVHLATIPITGT
InPIP1;2  TKGDGLGAEIVGTFILVYTVFSATDAKRNARDSHVPILAPLPIGFAVFLVHLATIPITGT
InPIP1;3  TKGDGLGAEIVGTFILVYTVFSATDAKRNARDSHVPVLAPLPIGFAVFLVHLATIPITGT
InPIP1;4  TKGDGLGAEIVGTFILVYVVFSATDAKRNARDSHVPILAPLPIGFAVFLVHLATIPITGT
InPIP2;1  SKGVGLSAEIIGTFVLVYTVFSATDPKRSARDSHVPVLAPLPIGFAVFMVHLATIPITGT
InPIP2;2  SRGTALGAEIIGTFVLVYTVFSATDPKRNARDSHIPVLAPLPIGFAVFMVHLATIPITGT
InPIP2;3  SKGVGLVAEILATFFLVYTVFSATDPKRNARDSHVPVLAPLPIGFAVFVAHVATIPITGT
InPIP2;4  NKGTGLGAEIIGTFVLVYTVFAATDPKRNARDSHVPVLAPLPIGFAVFMVHLATIPITGT
InPIP2;6  SKGTGLGAEIIGTFVLVYTVFSATDPKRNARDSHVPVLAPLPIGFAVFMVHLATIPVTGT
InPIP2;7  SKGTGLGAEIIGTFVLVYTVFAATDPKRNARDSHVPVLAPLPIGFAVFMVHLATIPITGT
InPIP2;8  SKGTGLGAEIIGTFVLVYTVFAATDPKRNARDSHVPVLAPLPIGFAVFMVHLATIPITGT
InPIP2;9  NKGTGLGAEIIGTFVLVYTVFSATDPKRNARDSHVPVLAPLPIGFAVFMVHLATIPITGT
```

```
              LE2
                                ____________________________   *  *
InPIP1;1  GINPARSLGAAIIYNNEHAWNDHWIFWVGPFVGAALAALYHQVVIRAIPFKSS-------
InPIP1;2  GINPARSLGAAIIYNKDDAWNDHWIFWVGPFIGAALAAVYHQIIIRAIPFKSRA------
InPIP1;3  GINPARSLGAAIIYNRDEAWNDHWIFWVGPFVGAALAALYHQVVIRAIPFKSS-------
InPIP1;4  GINPARSLGAAIIYNKDHAWDDQWIFWVGPFIGAALAALYHQVVIRAIPFKTGN------
InPIP2;1  GINPARSLGAAVIYNKDEAWHNHWIFWVGPFIGAAIAAFYHQFILRAGAAKALGSFRSGS
InPIP2;2  GINPARSFGAAVIYNNGKVWDEHWIFWVGPFVGALAAAAYHQYILRAAAIKALGSFRSNP
InPIP2;3  GINPARSFGAAVIHNNKHAWDDHWIFWVGPMLGAIAGAVYHQLVLRAQAVKALGGSFQST
InPIP2;4  GINPARSFGAAVIYNKDKAWDDQWIFWVGPFIGAFAAAVYHQFVLRASSIKALASFRSN-
InPIP2;5  GINPARSLGAAVMYNKDKAWNDHWIFWVGPFTGAAIAAFYHQFILRAGAVKAFGSFMSSS
InPIP2;6  GINPARSFGAAVIYGKDKAWDDQWIFWVGPFIGAAIAAIYHQFILRAGALKALGSQRSNA
InPIP2;7  GINPARSFGAAVIYGKDKAWDDQWIFWVGPFVGAAIAAVYHQYVLRAGAAKALGSYRSNA
InPIP2;8  GINPARSFGAAVIYGKDKAWDDQWIFWVGPFVGAAIAAVYHQYVLRAGAAKALGSYRSNA
InPIP2;9  GINPARSFGAAVIFNDDKAWDEHWIFWVGPFVGALAAAVYHQYILRGSAIKALGSFRSNA


InPIP1;1  ---
InPIP1;2  ---
InPIP1;3  ---
InPIP1;4  ---
InPIP2;1  QV-
InPIP2;2  TN-
InPIP2;3  PNV
InPIP2;4  ---
InPIP2;5  QV-
InPIP2;6  ---
InPIP2;7  ---
InPIP2;8  ---
InPIP2;9  ---
```

Figure S6: Alignment of AA sequences of InPIP subfamily members.

Shown is an AA sequence alignment of all InPIP. Black lines above the alignment indicate predicted transmembrane domains. The two conserved NPA motifs are shown in bold letters and marked in yellow. Residues comprising the ar/R filter are marked in blue and labelled H2, H5, LE1 and LE2. Residues occupying conserved positions one to five (from N- to C-terminus P1 to P5) are marked in green. Columns or regions with conserved putative phosphorylation sites are marked by an asterisk.
