# Supplementary material for: Genome-Wide Analysis of Aquaporins in Japanese Morning Glory (Ipomoea nil)
Source: Plants (Basel). 2023 Mar 30;12(7):1511. doi: 10.3390/plants12071511 (PMC10096635; doi:10.3390/plants12071511)
Supplement: Supplementary file 1 [file plants-12-01511-s001.zip › Figure S7.pdf]

```
InTIP1;1  -MPIHRIAIGAPAEASHPDALKAALAEFFSMLIFVFAGQGSGMAFSKLTD
InTIP1;2  -MPVRQIAIGRPAEATHPDALKAALAEFFSTLIFVFAGEGSGMAFAKLTG
InTIP1;3  -MPIRQVALGSHEELRHPGSLKAALAEFICTLIFVFAGQGSGVAYSKLTG
InTIP1;4  -MPIHQIAFGSHEELRQPGNLKAALAEFISTLIFVFAGQGSGMAFSKLTN
InTIP1;5  -MPIHRIAIGNPGEAAHPDALRAAFAEFFSTLIFVFAGQGSGMAFTKLTD
InTIP1;6  -MAVPRIAIGSVGEATQPDALKAAVAEFISMLIFVFAGSGSGMAFNKLTD
InTIP2;1  ---MPGIAFGRFDDSFSFASIKAYIAEFISTLLFVFAGVGSAIAFNKLTS
InTIP2;2  ---MPAIAFGRFDDSFSASSLKAYLAEFISTLLFVFAGVGSAIAYNKLTA
InTIP3;1  MAPPRRYAFGRADEATHPDSMRATLSEFLSTALFVFIGEGAVLAIDKLYR
InTIP4;1  ---MAKIALGNGSEALQSDCIQALIVEFICTFLFVFTGVGAAMATDQLQG
InTIP5;1  ----MASIRSRLEHCFTADALRAYLAEFISTMFFVFAAVGSSMSSRKMSP

                         H2
InTIP1;1  GGAATPAGLIAAALAHALALFVAVSVGANISGGHVNPAVTFGAFLGGNIT
InTIP1;2  GGPSTPSGLVAASIAHAFGLFVAVAVGANISGGHVNPAVTFGAFVGGNIT
InTIP1;3  DGASTPAGLISASIAHAFGLFVAVSVGANISGGHVNPAVTFGAFVGGNIT
InTIP1;4  DGASTPSGLIAAAIAHAFALFVAVSVGANISGGHVNPAVTFGAFVGGNIT
InTIP1;5  GGAPTPSGLIAAALSHGMALFVAVSVGANISGGHVNPAVTFGAFMGGHIT
InTIP1;6  NGAATPAGLISAAIAHAFALFVAVSVGANISGGHVNPAVTFGAFVGGHIT
InTIP2;1  DAALDAPGLVAIAVCHAFALFVAVSVGANISGGHVNPAVTFGLAVGGQIT
InTIP2;2  DAALDPAGLVAVAVCHGLALFVAVAIAANISGGHVNPAVTFGLAVGGQIT
InTIP3;1  DSALGASGLTVLALAHALALFAAVASSLNVSGGHVNPAVTFGALVGGRIS
InTIP4;1  N---ALVGLFFVAMAHALVVAVMISAGLRISGAHLNPAVTLGLLMGGHIT
InTIP5;1  EAASDPSSLVGIAVANAFALTVAVYISIGVSGGHVNPAVTFARAVGRHIS

InTIP1;1  LLRGILYWIAQLLGSVVACLLLKLATGGLETSAFALSSGVSVWNALIFEI
InTIP1;2  LLRGILYWIAQLLGSVAACLLLKFSTGGLETSGFGLSG-VGALNALVFEI
InTIP1;3  FFRGLLYVIAQLLGSTVACFLLELSTG-LPTGSFGLSG-VSVWSALVFEI
InTIP1;4  FFRGVLYVIAQLLGSTAACFLLEFATGGMSTGAFGLSG-VSVWSALIFEI
InTIP1;5  LLRSILYWIAQLLGSAIACLLLNYATGGMETSAFGLSSGVTTWNAVTFEI
InTIP1;6  LFRSVLYWIAQLLGSVVACLLLKFATGGLETPAFGLSAGVAPWNAVVFEI
InTIP2;1  ILTGLFYWIAQLLGSILACYLLTVVTGGLAVPTHSVAAGVGAVGGVVMEI
InTIP2;2  VITGLFYWVAQLLGSIVACYLLKIVTGGLAVPTHGVAAGVGAIEGVVMEI
InTIP3;1  FLLALYYWIAQLLGSIVACLLLRVSLDGMRPQGFSLAAGEGWGSGLLLEI
InTIP4;1  VIRSFLYLIDQLLASVAACALLTYLTGGLTTPAHTLASGVGYGQGVIMEI
InTIP5;1  LSMAIFYWISQLLGSVMACVLLKVFTVQQHVPVLGIPQEMTGFDAAILEG

                                                     H5        LE1
InTIP1;1  VMTFGLVYTVYATAVDPKKGDLGTIAPIAIGFIVGANILAGGAFDGASMN
InTIP1;2  VMTFGLVYTVYATAVDPKKGSLGTIAPIAIGFIVGANILAGGAFTGASMN
InTIP1;3  VMTFGLVYTVYATAIDPKKGDLGTIAPLAIGFIVGANILAGGAFTGASMN
InTIP1;4  VMTFGLVYTVYATAVDPKKGDLGTIAPIAIGFIVGANILAGGAFTGASMN
InTIP1;5  VMTFGLVYTVYATAVDPKKGDLGIIAPIAIGFIVGANILAGGAFDGASMN
InTIP1;6  VMTFGLVYTVYATAVDPKKGNIGIIAPIAIGFIVGANILAGGAFDGASMN
InTIP2;1  IITFGLVYTVYATAADPKKGSLGTIAPIAIGFIVGANILAAGPFSGGSMN
InTIP2;2  IITFALVYTVYATAVDPKKGSLGTIAPIAIGLIVGANILAAGPFSGGSMN
InTIP3;1  IMTFGLMYTVYATAIDPKRGSLGTIAPLAIAFIVGANVFVGAPFTGASMN
InTIP4;1  ILTFSLLFTVYATIVDPKKGNLDGLGPLLTGLVVGANIMAGGSFSGASMN
InTIP5;1  MMTFVLVYTVYAATD-PRKGALCAMGPLAIGMIAGGNVLASGAFTGGSMN
```

```
                LE2
InTIP1;1   PAVSFGPAVVSWTWTCHWVYWLGPLVGAAIAALVYDNIFIG---------
InTIP1;2   PAVAFGPAVVSWSWDAHWIYWVGPLVGGGIAGLVYDLIFIP---------
InTIP1;3   PAVAFGPSLVSWNWSCHWVYWLGPIVGGGLAGVVYELLFIG---------
InTIP1;4   PAVSFGPAVVSWTWTNHWVYWAGPIIGGGIAGVVYELFFIT---------
InTIP1;5   PAVAFGPAVVSWTWTHHWVYWLGPFLGAAIAALVYDNIFIG---------
InTIP1;6   PAVSFGPAVVSWSWECHWVYWLGPFLGAGIAALVYQVIFIC---------
InTIP2;1   PARSFGPAVVSGDFSSIWIYWVGPLVGGGLAGLIYPNVFMAH--------
InTIP2;2   PARSFGPAVVSLDFACNWIYWVGPLVGGGLAGLIYANVFMCH--------
InTIP3;1   PARAFGPALVGWRWRYHWIYWVGPFIGAGLAGLIYEFGILPPAADPPHTH
InTIP4;1   PARSFGPALVSGNWTDHWVYWVGPLIGGGLAGFIYETFFIVR--------
InTIP5;1   PAYAFGSALIGGNFGNQAAYWVGPLIGGTIAGVLYDKVVFPSESD-----

InTIP1;1   THHHEQLPIADY-
InTIP1;2   QHHEALP------
InTIP1;3   HPTHEPL------
InTIP1;4   HHTHEPLPRGEF-
InTIP1;5   GHPHEQLPN----
InTIP1;6   QNTHEQLPTTDY-
InTIP2;1   --EHAPLSSDF--
InTIP2;2   --EHAPLSSDF--
InTIP3;1   HTHHQPLAAEDY-
InTIP4;1   --THVPIATQEAF
InTIP5;1   DSTRPGLSEVGVV
```

Figure S7: Alignment of AA sequences of InTIP subfamily members.

Shown is an AA sequence alignment of all InTIP. Black lines above the alignment indicate predicted transmembrane domains. The two conserved NPA motifs are shown in bold letters and marked in yellow. Residues comprising the ar/R filter are marked in blue and labelled H2, H5, LE1 and LE2. Residues occupying conserved positions one to five (from N- to C-terminus P1 to P5) are marked in green.
