# Supplementary material for: Genome-Wide Analysis of Aquaporins in Japanese Morning Glory (Ipomoea nil)
Source: Plants (Basel). 2023 Mar 30;12(7):1511. doi: 10.3390/plants12071511 (PMC10096635; doi:10.3390/plants12071511)
Supplement: Supplementary file 1 [file plants-12-01511-s001.zip › Figure S8.pdf]

```
InSIP1;1  ---MGVIKAAIGDAVLTFLWVFSASTLGVSTSVLAKLLGIAHPMAVLSVT
InSIP2;1  MEGSRGLRMLASDFLMSLMWVWSS-VLIKVFVYGVLAFGDHDFHAEILK

                                                  H2
InSIP1;1  TVLFFILLFVFGIISDALGGASFNPTGIAAFYAAGLGDDSLISAAVRFPA
InSIP2;1  HTLAVAVMFFFAFLVNVTNGGAYNPLTVLASAISGDFKNFLFTVGARIPT

InSIP1;1  QAAGAVGGAMAILEVIPPHHKHMIIGPSLKVDLHTGAIAEGVLTFISSFI
InSIP2;1  QVLGSITGVRLILDTFP----DIGRGPKLNVDIHRGALTEGCLTFMIVII

                               H5            LE1    LE2
InSIP1;1  VFLVILKGPKNSFVKNWLLTIPTVVLVVAGSSYTGPSMNPANAFGWAYIN
InSIP2;1  SFGLSRQIPGSFFMKTWISSLSKLALHVLGSDLTGGCMNPASVMGWAYAR

InSIP1;1  NWHNTREQFYVYWICPFIGAILAAWMFRAIFPPPVKPKAKKN-----
InSIP2;1  GDHITKEHIQVYWLAPIQGTLLAVWIFRLMFPPQKEDKAKAKAEKSD
```

Figure S8: Alignment of AA sequences of InSIP subfamily members.

Shown is an AA sequence alignment of all InSIP. Black lines above the alignment indicate predicted transmembrane domains. The two conserved NPA motifs are shown in bold letters and marked in yellow. Residues comprising the ar/R filter are marked in blue and labelled H2, H5, LE1 and LE2. Residues occupying conserved positions one to five (from N- to C-terminus P1 to P5) are marked in green.
