# Supplementary material for: Genome-Wide Analysis of Aquaporins in Japanese Morning Glory (Ipomoea nil)
Source: Plants (Basel). 2023 Mar 30;12(7):1511. doi: 10.3390/plants12071511 (PMC10096635; doi:10.3390/plants12071511)
Supplement: Supplementary file 1 [file plants-12-01511-s001.zip › Figure S9.pdf]

```
InXIP1;1  --MAANLMHSFGDEESQTHSSRRCSRDDGAKG----------LSEEWTGKESKDDKPIFS
InXIP1;2  -MAAKYTRQGFGDEESHPHQNGGSNRVEDVSSTPTISNGNGNGTHQWTG--EKIPSSHLT
InXIP1;3  MAANTIPRHILGDEESHPHNIGGSKRIAAVFSPFPMG------DHEWTGDDQKTPPASVT

                                                              H2
InXIP1;1  LVQKLGLMDFVSLDVWRAAVAEVLGTAVLVFVLDTVVISTYETETKTPNLIMSILIAIFA
InXIP1;2  LAQKLGLMDFLSLDVWRASMGEVFGTAVLVFMLDAIVISTYESEVKMPNLIMSILIAIVI
InXIP1;3  LAQKLGVMDFFSLNVWRASIGELIGTAVLVFMIDAIVISTYETDIKMPNLVMSVLIAIVI

InXIP1;1  TIIILALLPISGGHVNPIISFSAALVGIISMSRAIIYILAQCIGAVLGALALKAVVSETI
InXIP1;2  TVLLLAVFPVSGGHINPTVTFSAALVGIISMSRAVIYMVAQCVGAVLGALALKAVVSSSI
InXIP1;3  TILLIAVFPVSGGHINPIISFSAALVGIISISRAVIYMVAQCVGATLGALALKAVVSSSI

InXIP1;1  QENFSLGGCTIRAVTQAPNGPVIAGLAIKQAFWMEFFCSFVLLFAVLWMAYDYRQSKALG
InXIP1;2  ENTFSLGGCTVTVVAPGPNGPVTVGLDTVQAFWLEVFCSFVFLFASIWMAYDYRQAKVLG
InXIP1;3  ENTFSLGGCTLTVIAPGPNGPITVGLETAQAFWLEIFCSFVFLFASIWMAYDHRQAKSLG

                              H5        LE1   LE2
InXIP1;1  QVKVFTFVGIVFGLLVFISTTVTKQKGYGGAGLNPARCLGAALVKGGHLWDGLWVFWVAP
InXIP1;2  HVKVFTVVGIVLGLLVFISTTLTTKKGYAGAGMNPARCVGAALVRGGHLWDGHWIFWVGP
InXIP1;3  LVKVLSIVGMVLGLLVFISTTVTAKKGYAGAGMNPARCFGAAVVRGGHLWDGHWIFWVGP

InXIP1;1  AIACMAFYLYTKIIPREHFHADGYPHDLFPLSK---------
InXIP1;2  LIACVAFYLYTKIIPERHFHASGYPHDFFSTVKVGLGIRPSI
InXIP1;3  FIACVAFYLYTKIIPTQHFHASGYPHDFFSTLKLACGSPQL-
```

Figure S9: Alignment of AA sequences of InXIP subfamily members.

Shown is an AA sequence alignment of all InXIP. Black lines above the alignment indicate predicted transmembrane domains. The two conserved NPA motifs are shown in bold letters and marked in yellow. Residues comprising the ar/R filter are marked in blue and labelled H2, H5, LE1 and LE2. Residues occupying conserved positions one to five (from N- to C-terminus P1 to P5) are marked in green.
