# Supplementary material for: Genome-Wide Analysis of Aquaporins in Japanese Morning Glory (Ipomoea nil)
Source: Plants (Basel). 2023 Mar 30;12(7):1511. doi: 10.3390/plants12071511 (PMC10096635; doi:10.3390/plants12071511)
Supplement: Supplementary file 1 [file plants-12-01511-s001.zip › Table S1.pdf]

Table S1. Homology of AQP in morning glory with AQP in Arabidopsis and tomato

Name	Gene ID	Homolog	The percent homology	Query coverage	e-value
InPIP1;1	INIL02g16994	AtPIP1;1	82%	1 - 284	e-134
InPIP1;2	INIL05g23971	AtPIP1;1	82%	2 - 286	e-139
InPIP1;3	INIL05g24206	AtPIP1;1	81%	1 - 284	e-135
InPIP1;4	INIL09g29995	AtPIP1;1	85%	1 - 284	e-139
InPIP2;1	INIL03g18022	AtPIP2;1	79%	1 - 287	e-127
InPIP2;2	INIL04g34732	AtPIP2;1	73%	3 - 283	e-116
InPIP2;3	INIL05g09496	AtPIP2;1	68%	13 - 287	e-104
InPIP2;4	INIL08g34668	AtPIP2;1	76%	1 - 283	e-122
InPIP2;5	INIL09g21386	AtPIP2;1	73%	1 - 287	e-120
InPIP2;6	INIL10g11305	AtPIP2;1	78%	1 - 285	e-126
InPIP2;7	INIL12g15802	AtPIP2;1	77%	1 - 285	e-125
InPIP2;8	INIL12g15803	AtPIP2;1	77%	1 - 285	e-125
InPIP2;9	INIL12g22089	AtPIP2;1	77%	1 - 285	e-127
InTIP1;1	INIL02g16891	AtTIP1;1	80%	1 - 251	e-101
InTIP1;2	INIL04g02479	AtTIP1;1	82%	1 - 247	e-112
InTIP1;3	INIL07g08709	AtTIP1;1	73%	1 - 246	5.00e-97
InTIP1;4	INIL08g00150	AtTIP1;1	71%	1 - 251	7.00e-89
InTIP1;5	INIL11g18467	AtTIP1;1	75%	1 - 248	e-102
InTIP1;6	INIL12g22155	AtTIP1;1	79%	1 - 251	e-105
InTIP2;1	INIL01g19987	AtTIP2;1	78%	1 - 250	2.00e-99
InTIP2;2	INIL03g18342	AtTIP2;1	79%	1 - 250	e-104
InTIP3;1	INIL03g01865	AtTIP3;1	48%	7 - 136	6.00e-26
InTIP4;1	INIL14g06843	AtTIP4;1	71%	1 - 244	1.00e-94
InTIP5;1	INIL02g17136	AtTIP5;1	57%	8 - 251	2.00e-75
InNIP1;1	INIL06g35686	AtNIP1;1	61%	1 - 290	6.00e-98
InNIP1;2	INIL14g02021	AtNIP1;1	59%	50 - 263	2.00e-74
InNIP1;3	INIL14g02022	AtNIP1;1	53%	11 - 277	1.00e-82
InNIP2;1	INIL00g00315	AtNIP2;1	40%	45 - 263	8.00e-44
InNIP2;2	INIL00g18375	AtNIP2;1	34%	39 - 263	2.00e-33
InNIP2;3	INIL11g18696	AtNIP2;1	37%	45 - 195	1.00e-23
InNIP3;1	INIL14g41661	AtNIP3;1	46%	39 - 268	7.00e-57
InNIP4;1	INIL02g40633	AtNIP4;1	52%	1 - 270	5.00e-76
InNIP4;2	INIL06g37831	AtNIP4;1	48%	43 - 268	6.00e-59
InNIP4;3	INIL08g31098	AtNIP4;1	55%	8 - 206	1.00e-57
InNIP5;1	INIL05g21764	AtNIP5;1	78%	29 - 304	e-123
InNIP5;2	INIL06g37646	AtNIP5;1	77%	29 - 240	3.00e-88
InNIP5;3	INIL15g31300	AtNIP5;1	76%	80 - 293	4.00e-91
InNIP6;1	INIL09g36261	AtNIP6;1	75%	90 - 305	4.00e-88
InNIP7;1	INIL13g08057	AtNIP7;1	52%	48 - 262	1.00e-57
InSIP1;1	INIL04g32909	AtSIP1;1	44%	2 - 95	2.00e-12
	+				
	INIL04g32910	AtSIP1;1	50%	119 - 227	4.00e-23
InSIP2;1	INIL04g32936	AtSIP2;1	55%	6 - 237	8.00e-77
InXIP1;1	INIL04g34767	SlXIP1;1	56%	1 - 322	e-104
InXIP1;2	INIL06g38432	SlXIP1;1	65%	9 - 322	e-119
InXIP1;3	INIL06g38434	SlXIP1;1	63%	1 - 322	e-116
